# Carnosine Prevents Type 2 Diabetes-Induced Osteoarthritis Through the ROS/NF-κB Pathway

**DOI:** 10.3389/fphar.2018.00598

**Published:** 2018-06-06

**Authors:** Yue Yang, Yang Wang, Yawei Kong, Xiaoning Zhang, He Zhang, Yi Gang, Lunhao Bai

**Affiliations:** ^1^Department of Orthopedic Surgery, Shengjing Hospital of China Medical University, Shenyang, China; ^2^Department of Ultrasound, Shengjing Hospital of China Medical University, Shenyang, China; ^3^International Patient Center, Brigham and Women’s Hospital, Harvard Medical School, Boston, MA, United States

**Keywords:** carnosine, osteoarthritis, type 2 diabetes mellitus, fibroblast-like synoviocyte, reactive oxygen species, NF-kappaB

## Abstract

**Background:** The anti-inflammatory and antioxidant capacity of carnosine (CAR) has been investigated in autoimmune diseases. The aim of this study was to evaluate the potential protective effects of oral CAR supplements to ameliorate type 2 diabetes mellitus (T2DM)-induced osteoarthritis (OA) in rats and its mechanism.

**Methods:** Seventy male Sprague–Dawley rats were randomly divided into the control group (CG, *n* = 10) and the T2DM group (*n* = 60). A rat model of T2DM was established using a high fat diet and streptozotocin (30 mg/kg, i.p.). The 41 rats that developed T2DM were chosen and randomly divided into four groups: T2DM-induced OA group (OAG, *n* = 11), and the T2DM-induced OA with low, moderate, and high-doses of CAR for 8 weeks group (CAR-L, CAR-M, and CAR-H, *n* = 10). After 13 weeks, all rats were evaluated by enzyme-linked immunosorbent assay (ELISA), histology, immunohistochemistry, and western blotting. Fibroblast-like synoviocytes (FLSs) were obtained from the knee joints of all rats. The effects of CAR on the inflammatory response in interleukin (IL)-1β-stimulated FLSs under a high glucose environment were evaluated by real-time quantitative polymerase chain reaction, western blotting, flow cytometry, and immunofluorescence.

**Results:** The results of ELISA (IL-1β and tumor necrosis factor-α), the histological evaluation (Mankin and OARSI score), western blotting [COL2A1, matrix metalloproteinase (MMP)-3, MMP-13, IL-1β, and nuclear factor-kappaB (NF-κB) p65], and immunohistochemistry (COL2A1, MMP-3, and MMP-13) indicated that oral CAR attenuated the development of T2DM-induced OA and suppressed the inflammatory response. Moreover, CAR alleviated MMP-3 and MMP-13 expression levels by decreasing reactive oxygen species content and suppressing nuclear translocation of NF-κB p65 on IL-1β-induced FLSs in a high glucose environment.

**Conclusion:** These findings indicate that oral CAR had chondroprotective effects on T2DM-induced OA through the reactive oxygen species (ROS)/NF-κB pathway.

## Introduction

Knee osteoarthritis (OA) is a highly prevalent disabling joint disease that is poorly understood and it has doubled in prevalence since the mid-20th century (Ondrésik et al., 2017; Wallace et al., 2017). OA involves the whole joint, including degeneration of the articular cartilage, subchondral bone porosis, and inflammation of the synovial membranes (Kalunian, 2016). Although many clinical therapies for OA focus on relieving symptoms, reducing pain, and improving joint function, the pathophysiological processes need further investigation.

Type 2 diabetes mellitus (T2DM) is characterized by reduced pancreatic β-cell function and systemic insulin resistance, which lead to metabolic dysfunction throughout the body. OA and T2DM often co-exist and share many risk factors, including aging, obesity, and physical inactivity (Sellam and Berenbaum, 2013; Guariguata et al., 2014; King and Rosenthal, 2015). T2DM can develop with OA in association with high fat diet-induced obesity, glucose intolerance, and insulin resistance in a classic rat model (Mooney et al., 2011; King and Rosenthal, 2015; Hamada et al., 2016; Williams et al., 2016).

The association between T2DM and OA involves chronic systemic inflammation related to metabolic syndrome (Dahaghin et al., 2007; Brunner et al., 2012; Gierman et al., 2012; Griffin et al., 2012) The cartilage degradation that occurs in OA results from dysregulated joint homeostasis activated by pro-inflammatory mediators, such as cytokines, lipid mediators, and reactive oxygen species (ROS), which are produced by synoviocytes (de Boer et al., 2012; Berenbaum et al., 2013; Kirkman, 2015). Adipokines act on the synovial membrane of the joint to increase the number of activated macrophages that release pro-inflammatory cytokines, such as interleukin-1β (IL-1β), tumor necrosis factor-α (TNF-α), and ROS (de Boer et al., 2012; Lee et al., 2013). ROS are a major contributor to chronic low-grade inflammation and are excessively generated by hyperglycemia-induced oxidative stress, which is a result of an imbalance between the peroxidation and antioxidant defense systems (Shuai et al., 2013).

Synovitis is the main manifestation of OA. The synovium consists of synovial macrophages and fibroblast-like synoviocytes (FLSs). FLSs are the key cells mediating the destruction of cartilage in OA (Kalunian, 2016). FLSs are activated by pro-inflammatory cytokines and cytokine-independent pathways, including IL-1β. We examined the expression of matrix metalloproteinase (MMP)-3 and MMP-13, as elevated levels of MMPs, mainly secreted by FLSs, may be the main cause of OA. The nuclear factor kappaB (NF-κB) pathway orchestrates mechanical, inflammatory, and oxidative stress-activated processes, thus representing a potential therapeutic target in OA disease (Olivotto et al., 2015; Yang et al., 2017).

Although many pharmacological agents are available to relieve OA symptoms, these agents, such as non-steroidal anti-inflammatory drugs, are associated with substantial gastrointestinal, renal, and cardiovascular side effects (Patrignani et al., 2011; Cheng and Visco, 2012). Thus, the development of novel therapeutic agents that can ameliorate OA damage and be safe to administer for long periods is urgently needed. Amino acids also play an important role in ameliorating T2DM and arthritis, such as methionine (Li et al., 2016), arginine (Clemmensen et al., 2013), and so on. Carnosine (CAR) is a dipeptide consisting of β-alanine and L-histidine. The anti-inflammatory potential of CAR in autoimmune diseases has been investigated (Ponist et al., 2016). The capacity of CAR is well documented, such as antioxidant, anti-glycating, aldehyde-scavenging, and toxic metal-ion chelating properties (Fatih et al., 2017). CAR suppresses senescence of cultured human fibroblasts and delays aging, but the mechanisms remain uncertain (Hipkiss, 2009). Nevertheless, the anti-inflammatory potential of CAR in OA has been scarcely investigated.

In the present study, we evaluated the potential protective effects of an oral CAR supplement to ameliorate T2DM-induced OA. Furthermore, to elucidate a potential contribution by an anti-inflammatory mechanism to these effects at the cellular level, we explored the therapeutic effects of CAR focusing on the ROS/NF-κB signaling pathway in IL-1β-induced FLSs under a high glucose condition.

## Materials and Methods

### Experimental Animals

Seventy male Sprague–Dawley (SD) rats (130–140 g, and specific-pathogen-free) were obtained from HFK Bioscience Co., Ltd. (Beijing, China). This study was carried out in accordance with the recommendations of “the Ethics Committee of Shengjing Hospital of China Medical University.” The protocol was approved by this committee. The rats were kept in individual plastic cages on sawdust bedding, under a 12:12 h light: dark cycle with lights on from 6:00 a.m. to 6:00 p.m., a controlled temperature of 22 ± 2°C, and 70% humidity. The rats had free access to a planned diet. Body weight was recorded at regular intervals. The rats were adapted to laboratory conditions for 1 week prior to the experimental procedures.

### T2DM Model and Oral CAR Supplementation

After the 1 week acclimation, the rats were randomly assigned to a control group (CG, *n* = 10) or a T2DM group (*n* = 60). In the CG, rats were fed a normal chow diet (10% of kcal derived from fat). T2DM was induced with a combination of a high-fat diet (60% kcal derived from fat) and streptozotocin (STZ) treatment (Reed et al., 2000; Srinivasan et al., 2005; Liu et al., 2012). In the T2DM group, the rats were first fed a high-fat diet for 4 weeks. Then, the rats were fasted the night before drug administration and injected i.p. with a single dose of STZ (Srinivasan et al., 2005; Deeds et al., 2011; Sigma, Beijing, China, 30 mg/kg body weight) dissolved in citrate buffer (0.1 M, pH 4.4). The CG group received citrate buffer only. Seventy-two hours after the STZ injection, glucose was measured in blood samples, obtained by a tail prick and a strip-operated blood glucose sensor (Onetouch Ultraeasy; Ningbo Qihao International Trade Co., Ltd., Ningbo, China). Blood glucose levels were >16.7 mmol/L in all STZ-injected animals and defined as diabetic rats for further pharmacological studies (Srinivasan et al., 2005; Shi et al., 2011).

**FIGURE 1:**
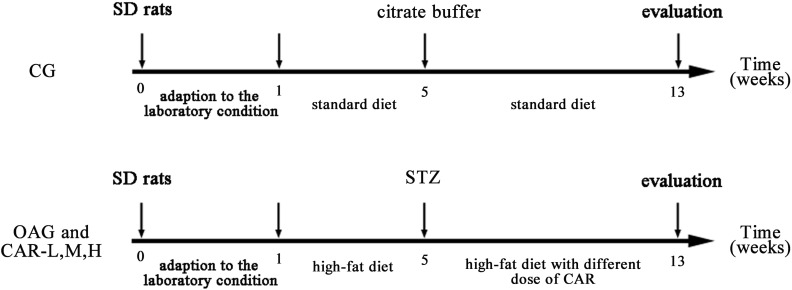
Treatment schedule and intervals for various parameters.

After the STZ injection, the CG was kept on the standard diet, and the T2DM group was kept on the high-fat diet. A total of 41 rats that developed T2DM were chosen and randomly divided into four treatment groups: T2DM-induced OA group (OAG, *n* = 11), T2DM-induced OA + low-dose CAR supplement group: 0.1 g/kg/day for 8 weeks (CAR-L, *n* = 10); T2DM-induced OA + moderate-dose CAR supplement group: 0.3 g/kg/day for 8 weeks (CAR-M, *n* = 10); and T2DM-induced OA + high-dose CAR supplement group: 0.9 g/kg/day for 8 weeks (CAR-H, *n* = 10; **Figure 1**).

### Sampling and Tissue Preparation

All rats were anesthetized after oral CAR supplementation. Blood samples were obtained immediately after the animals were euthanized and centrifuged at 3000 × *g* for 10 min to obtain the serum. All rats were sacrificed by cervical dislocation. Intra-articular lavage fluid (IALF) was obtained from the synovial cavity of the right knee by injecting 200 μl of phosphate-buffered saline (PBS) three times, followed by recovery of the liquid. The left knee joints of all rats were dissected and fixed in 4% paraformaldehyde solution. The articular cartilage and synovium were removed from the weight-bearing areas of the condyles of the right femur and tibia using a scalpel. All tissues were stored at -80°C.

### Histology

Left knee joint tissue samples were stored in 4% paraformaldehyde for 7 days. Then, they were washed in water for 5 h and transferred to 20% EDTA solution (Jianglai Reagent Co., Ltd., Shanghai, China) to decalcify for 21 days; the solution was changed every 3 days. Decalcified samples were dehydrated in an ethanol series and embedded in paraffin. Serial 5-μm sagittal sections were cut from the tibiofemoral joints for histological examination. The sections were stained with hematoxylin and eosin, as well as toluidine blue, to observe the cartilage. Next, the sections were visualized with ScanScope (APERIO CS2, Leica Biosystems, Inc., Buffalo Grove, IL, United States). Injuries to the articular cartilage in the femur and tibia were assessed by the Modified Mankin score (scale of 0–14 points; van der Sluijs et al., 1992; Patrignani et al., 2011) and the Osteoarthritis Research Society International (OARSI) score (scale of 0–24 points; Pritzker et al., 2006; Gerwin et al., 2010; Ponist et al., 2016). Since both the tibial and femoral cartilages were evaluated, the maximum Mankin score was 28 and the maximum OARSI score was 48. Two experienced observers (YY and XZ) performed the scoring in a blinded manner.

### Measurement of Plasma Glucose, Total Cholesterol, and Triglycerides

Plasma glucose was determined by a strip-operated blood glucose sensor (Onetouch Ultraeasy; Ningbo Qihao International Trade Co., Ltd.). Plasma total cholesterol and triglycerides were measured using commercially available colorimetric diagnostic kits according to the manufacturer’s instructions (Bio-Technology and Science Inc., Beijing, China).

### Enzyme-Linked Immunoassay (ELISA) of TNF-α and IL-1β

Serum and knee IALF TNF-α and IL-1β levels were determined using enzyme-linked immunosorbent assay (ELISA) kits (Tongwei, Shanghai, China) following the manufacturer’s instructions. Then, the protein content in the IALF was measured to ensure that the ratio of the dilution was equal.

### Immunohistochemistry

In addition to the histomorphological evaluation, serial sections were stained for COL2A1, MMP-3, and MMP-13. After deparaffinization and rehydration of the tissue sections, the proteins were immunostained using a two-step method following the manufacturer’s instructions for the kit. The sections were incubated with rabbit polyclonal anti-COL2A1 antibody (ab34712, 1:50; Abcam, Cambridge, MA, United States), rabbit polyclonal anti-MMP-3 antibody (ab52915, 1:50; Abcam), and rabbit polyclonal anti-MMP-13 antibody (ab39012, 1:50; Abcam) overnight at 4°C. The slides were washed three times in PBS followed by a 20 min incubation at 37°C with an anti-mouse/rabbit IgG detection system (PV-9000, Zhongshan Goldenbridge Biotechnology Co., Beijing, China) and visualized with diaminobenzidine. Nuclei were counterstained with hematoxylin for 5 min. Negative control sections were prepared using the same protocol, but the primary antibody was replaced with PBS. The optical density of the stained slides was measured using image analysis software (NikonH600L microscope and image analysis system, Tokyo, Japan). COL2A1 and MMP-3 were expressed as relative intensities. MMP-13 was expressed as a percentage of positive cells.

### Western Blotting

The cartilage and synovium were washed twice in ice-cold PBS. Proteins in the cytoplasm and nucleus were isolated by using Cytoplasmic and Nuclear Protein Extraction Kit (AR0106, Boster, China), according to the manufacturer’s instruction. The protein concentration of cytoplasm and nuclear was measured with a bicinchoninic acid (BCA) assay kit (P0010S, Beyotime, China). Equal amounts of protein (40 μg) were separated by sodium dodecyl-sulfate-polyacrylamide gel electrophoresis and transferred to polyvinylidene difluoride membranes. After blocking with 1% bovine serum albumin (BSA) in Tris-buffered saline with 0.1% Tween-20 (TBST) at room temperature for 2 h, the blots were incubated overnight at 4°C with primary antibodies: rabbit polyclonal anti-COL2A1 antibody (ab34712, 1:5000; abcam), molecular weight 142 kDa; rabbit polyclonal anti-MMP-13 antibody (ab39012, 1:3000; abcam), molecular weight 54 kDa; rabbit monoclonal anti-MMP-3 antibody (ab52915, 1:1000; abcam), molecular weight 54 kDa; rabbit polyclonal anti-IL-1β antibody (ab150777, 1:1000; abcam), molecular weight 31 kDa; rabbit monoclonal anti-IκB-α antibody (ab32518, 1:10000; abcam), molecular weight 36 kDa; rabbit polyclonal anti-NF-κB p65 antibody (AB21014, 1:1000; absci), molecular weight 65 kDa; mouse monoclonal anti-β-actin (60008-1-lg, 1:5000, Proteintech Group), molecular weight 42 kDa; and rabbit polyclonal anti-histone H2A.X (AB41012, 1:1000, absci), molecular weight 19 kDa. After washing three times with TBST, the membranes were incubated with IgG-horseradish peroxidase-conjugated secondary antibodies (1:10,000, Canlife) at room temperature for 2 h. After washing with TBST buffer, immunoreactivity was detected with enhanced chemiluminescence and quantified using Quantity ONE (Bio-Rad, Hercules, CA, United States) software. β-actin or histone H2A.X was used as the internal control.

### Isolation and Culture of FLSs

Fibroblast-like synoviocytes were obtained from the knee joint synovium of SD rats. The tissues were collected in sterile PBS. Fat and connective tissue were removed, and the remaining tissue was digested with 1 mg/ml collagenase (Sigma–Aldrich, St. Louis, MO, United States) for 45 min at 37°C. The cells were then separated from the undigested tissue using a 70 μm cell strainer and cultured in 25 cm^2^ cell culture flasks in Dulbecco modified Eagle medium (Gibco BRL, Grand Island, NY, United States) with 10% fetal bovine serum (Gibco BRL) and antibiotics (100 U/ml penicillin and 100 μg/ml streptomycin) in a humid atmosphere of 5% CO_2_ at 37°C. Upon reaching confluence, the cells were detached with 0.25% trypsin and split 1:3. The cells were identified by immunofluorescence staining with vimentin antibody (ab92547, 1:200; Abcam; **Supplementary Figure S1**). Passages 4–6 were used for all experiments. More than 95% of the cells were judged to be FLSs under a microscope.

### Quantitative Real-Time Polymerase Chain Reaction (qPCR)

Cultured FLSs were grown in 100 mm cell culture dishes (8–10 × 10^6^ cells/dish) for quantitative polymerase chain reaction (qPCR). After the indicated treatment, the cells were washed twice with ice-cold PBS, and total mRNA was extracted with Trizol reagent. cDNA was reverse transcribed from 1 μg total RNA using a PrimeScript RT reagent kit with the gDNA Eraser (Takara Bio, Dalian, China) according to the manufacturer’s instructions. qPCR was performed in an ABI Prism 7500 Fast Real-Time PCR System (Applied Biosystems, Wilmington, NC, United States) using SYBR Premix Ex Taq II (Tli RNaseH Plus; Takara Bio, Dalian, China). Expression levels were calculated by the 2^-ΔΔ^CT method (Livak and Schmittgen, 2001) with β-actin as the reference gene. The primer pair sequences were specific to rat MMP-13 (F-5′-TGATGATGAAACCTGGACAAGCA-3′; R-5′-GAACGTCATCTCTGGGAGCA-3′), MMP-3 (F-5′-CATAATACACAGCTGACCTGTATAA-3′; R-5′-ATTTAAGAAATCATAGATAACAGTTACTTA-3′), and β-actin (F-5′GGAGATTACTGCCCTGGCTCCTA-3′; R-5′-GACTCATCGTACTC CTGCTTGCTG-3′).

### Treatment of FLSs

After the FLSs were cultured in six-well plates (2 × 10^6^ cells/well), they were stimulated with 10 ng/ml IL-1β (ab200284, Abcam) with or without CAR (100 μM) for 24 h under normal glucose (5.5 mM) or high glucose (25 mM) conditions. The CAR dose was considered to be effective after a dose–effect experiment (**Figure 6A**).

To investigate whether the effect of high glucose was related to its osmotic effect, cells were incubated with mannitol (19.5 mM) instead of high glucose (25 mM) for 24 h in separate experiments.

### Cellular ROS Production

Fibroblast-like synoviocytes were seeded and cultured in six-well plates at a density of 2 × 10^6^ cells per well. After 24 h, ROS production was measured by flow cytometry and fluorescence microscopy with 2′,7′-dichlorodihydrofluorescein diacetate (DCFH-DA) (S0033, Beyotime). The FLSs were incubated with 10 μM DCFH-DA for 45 min at 37°C in the dark, and then washed three times in PBS. Fluorescence was detected by fluorescent microscopy and was measured with the BD FACSCalibur at 488 nm excitation and 525 nm emission wavelengths.

### Western Blot and Immunofluorescence Analysis of FLSs

Cultured FLSs were grown in 100 mm cell culture dishes (8–10 × 10^6^ cells/dish) for the western blot analysis. FLSs were stimulated with 10 ng/ml IL-1β with or without CAR (100 μM) for 24 h under normal glucose (5.5 mM) or high glucose (25 mM) conditions. After 24 h, the cells were collected and stored at -80°C for western blotting.

Fibroblast-like synoviocytes were placed on a confocal dish and incubated under different conditions for 24 h. The cells were washed in PBS and fixed in 4% paraformaldehyde for 20 min at room temperature. Then, the cells were permeabilized with 0.5% Triton X-100 for 30 min after being incubated in non-specific binding blocking solution (5% BSA) for 30 min at room temperature. Rabbit polyclonal anti-NF-κB p65 antibody (AB21014, 1:50; Ab Science, Chatham, NJ, United States) was added to cells overnight at 4°C followed by staining with AlexaFluor^®^ 594 conjugated anti-rabbit antibody for 60 min at room temperature in the dark. Nuclei were counterstained with 4,6-diamidino-2-phenylindole (DAPI) for 2 min. After washing, the FLSs were visualized under a confocal microscope (Olympus, Tokyo, Japan).

### Statistical Analysis

Data are expressed as means with 95% confidence intervals (CIs) and analyzed using SPSS statistical software version 16 (SSPS, Inc., Chicago, IL, United States). The Shapiro–Wilk and Levene tests were applied to evaluate the normality and homogeneity of the results, respectively. One-way analysis of variance was used for the statistical analysis if the variables were normally distributed. *P*-values < 0.05 were considered significant.

## Results

### Histological Observations

**FIGURE 2:**
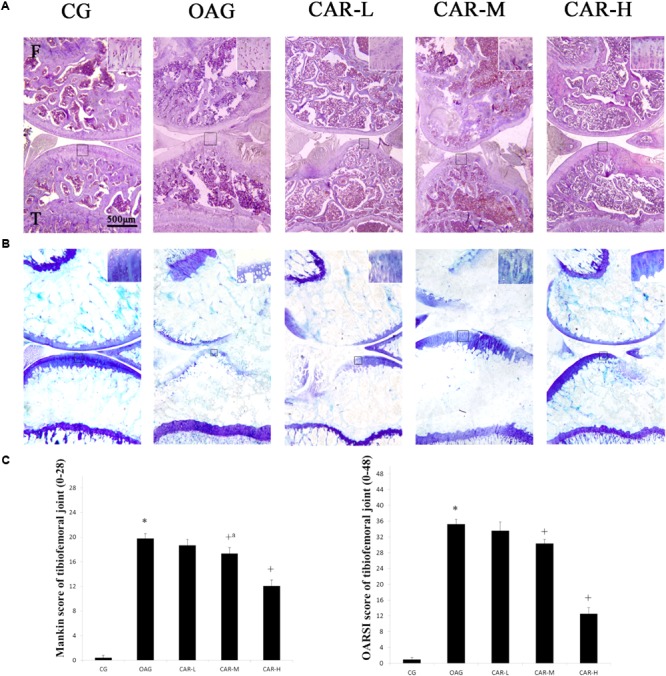
Histological evaluation of the tibiofemoral joints. **(A)** Histological features of representative tibiofemoral joints sectioned in the sagittal plane stained with hematoxylin and eosin and toluidine blue. Mankin and OARSI histological scores are shown for each image. F, femur; T, tibia. **(B)** Mankin score for the tibiofemoral joints. Differences between CG and OAG (^∗^*P* < 0.001), OAG vs. CAR-M and CAR-H groups (^+a^*P* = 0.002, ^+^*P* < 0.001) were significant. **(C)** OARSI histological scores for tibiofemoral joint cartilage. Differences between CG and OAG (^∗^*P* < 0.001), OAG vs. CAR-L, CAR-M, and CAR-H groups (^+^*P* < 0.001) were significant. One-way ANOVA, *n* = 10 or 11 rats in each group; means with 95% confidence intervals.

The histological assessment (Mankin and OARSI scores) demonstrated that the damage to the cartilage in the OAG was serious compared with that observed in the CG (Mankin score of tibiofemoral joints: CG = 1.0, 95% CI 0.6–1.4; OAG = 19.8, 95% CI 19.1–20.5; OARSI score of tibiofemoral joints: CG = 1.0, 95% CI 0.6–1.4; OAG = 35.3, 95% CI 34.2–36.4). CAR had therapeutic effects on the cartilage in the tibiofemoral joints compared with the OAG (Mankin score of tibiofemoral joints: CAR-L = 18.7 – 95% CI 17.8–19.6; CAR-M = 17.4 – 95% CI 16.4–18.3; CAR-H = 12.1 – 95% CI 11.2–13.0; OARSI score of tibiofemoral joints: CAR-L = 33.6 – 95% CI 31.4–35.8; CAR-M = 30.4 – 95% CI 29.3–31.4; CAR-H = 12.5 – 95% CI 11.0–14.1; **Figures 2A–C**).

### Results of Plasma Glucose, Total Cholesterol, and Triglycerides

**FIGURE 3:**
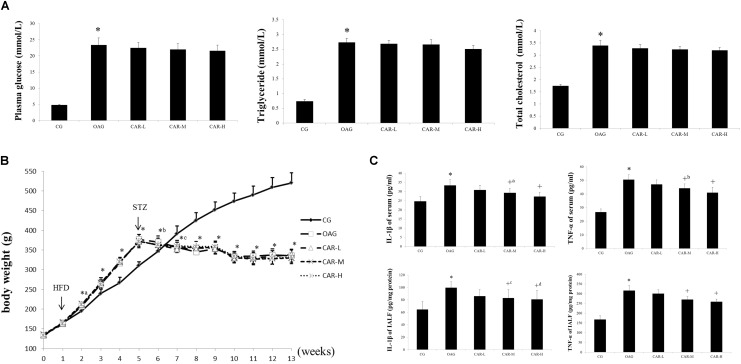
The results of body weight, serum, and intra-articular lavage fluid (IALF). **(A)** The results of plasma glucose, triglycerides, and total cholesterol. The differences between the CG and OAG were significant (^∗^*P* < 0.001), but no significant differences were observed between OAG and CAR-L, CAR-M, or CAR-H. **(B)** Body weights. Differences between the CG and OAG were significant (^∗^*P* < 0.001, ^∗a^*P* = 0.002, ^∗b^*P* = 0.030, and ^∗c^*P* = 0.001), but no significant differences were observed between OAG and CAR-L, CAR-M, or CAR-H. **(C)** Serum and IALF IL-1β and TNF-α levels. Differences between the CG and OAG were significant (^∗^*P* < 0.001), and differences between the OAG group and CAR-L, CAR-M, and CAR-H were significant (^+^*P* < 0.001, ^+a^*P* = 0.013, ^+b^*P* = 0.002, ^+c^*P* = 0.022, and ^+d^*P* = 0.011). One-way ANOVA, *n* = 10 or 11 rats for each group; means with 95% confidence intervals.

As shown in **Figure 3A**, the concentrations of plasma glucose, total cholesterol, and triglycerides increased in the OAG compared to the CG; but no significant differences were observed between the OAG and CAR-L, CAR-M, or CAR-H.

### ELISA for TNF-α and IL-1β

As shown in **Figure 3C**, serum TNF-α and IL-1β concentrations were both higher in the OAG than in the CG; CAR-M and CAR-H reduced the increase in serum TNF-α and IL-1β concentrations compared to that seen in the OAG group. The changes in the TNF-α and IL-1β concentrations were similar in IALF to those observed in serum (**Figure 3C**).

### Immunohistochemical Analysis

**FIGURE 4:**
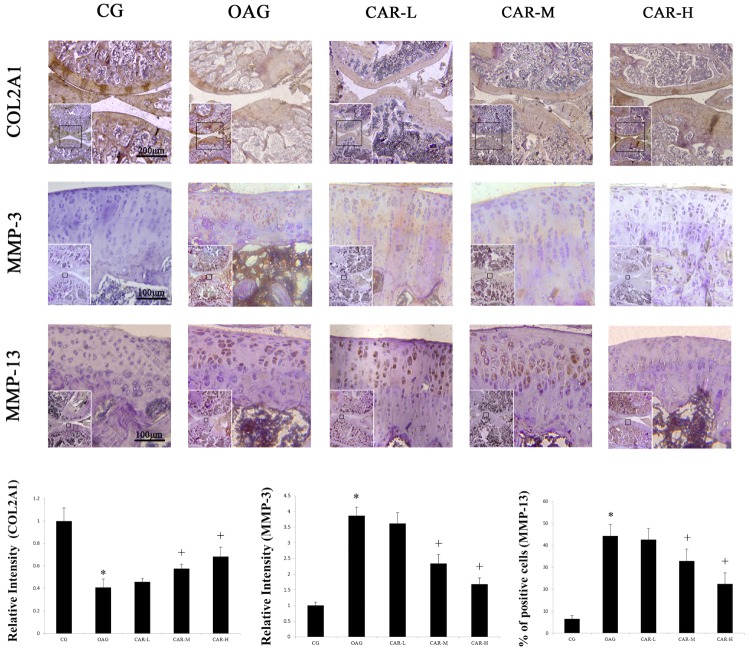
Immunohistochemical staining. The micrographs show the relative intensity of immunohistochemical staining for COL2A1 and MMP-3, and the percentages of MMP-13 positively stained cells in the articular cartilage of each experimental group. Differences between the CG and OAG were significant (^∗^*P* < 0.001), and differences between the OAG vs. CAR-M and CAR-H were significant (^+^*P* < 0.001). One-way ANOVA, *n* = 5 rats for each group, mean score with 95% confidence intervals.

The immunohistochemical staining showed that the therapeutic effects of CAR-M and CAR-H resulted in an increase of COL2A1, MMP-3, and MMP-13 expression in articular cartilage compared with the OAG group (**Figure 4**).

The relative intensity of COL2A1 compared with CG in the articular cartilage increased from 0.41 in the OAG group to 0.46 in the CAR-L, 0.58 in the CAR-M, and 0.69 in the CAR-H. The relative intensity of MMP-3 compared with CG in the articular cartilage decreased from 3.86 in the OAG group to 3.62 in the CAR-L, 2.34 in the CAR-M, and 1.68 in the CAR-H. The percentage of MMP-13 in the CG group was 6.6%. The percentage of MMP-13-positive cells in articular cartilage decreased from 44.3% in the OAG to 52.6% in the CAR-L, 32.9% in the CAR-M, and 22.4% in the CAR-H.

### Western Blot Analysis

**FIGURE 5:**
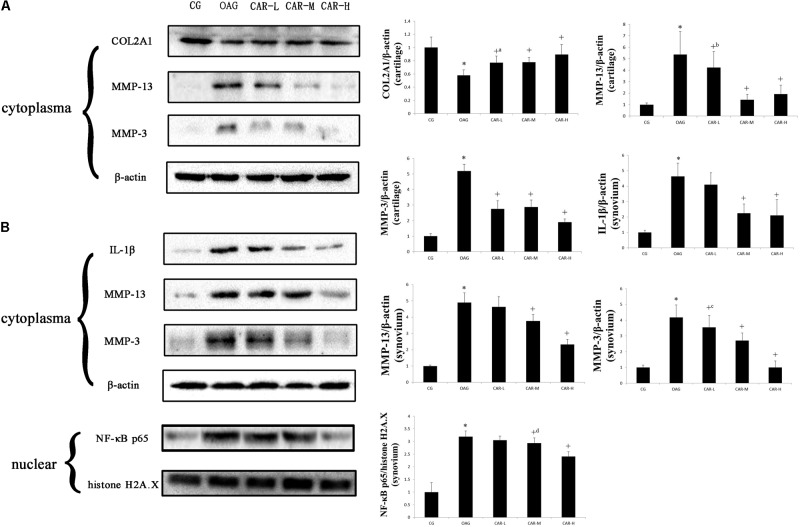
Western blotting results. Protein expression was determined in western blots of total protein extracted from cartilage **(A)** and synovium **(B)**. The data were obtained from three separate experiments β-actin or histone H2A.X was the internal standard. Differences between the CG and OAG group were significant (^∗^*P* < 0.001), and differences between the OAG and the different doses of CAR were significant (^+^*P* < 0.001, ^+a^*P* = 0.001, ^+b^*P* = 0.014, ^+c^*P* = 0.008, and ^+d^*P* = 0.010). One-way ANOVA, *n* = 3 rats for each group; means with 95% confidence intervals.

Western blots were evaluated for differences in COL2A1, MMP-13, and MMP-3 in cartilage and IL-1β, MMP-13, MMP-3, and NF-κB p65 in the synovium among the groups of rats with T2DM-induced knee OA (**Figure 5**). Treatment with CAR resulted in these proteins being expressed in articular cartilage and synovium compared with the OAG group.

### qPCR Assay

**FIGURE 6:**
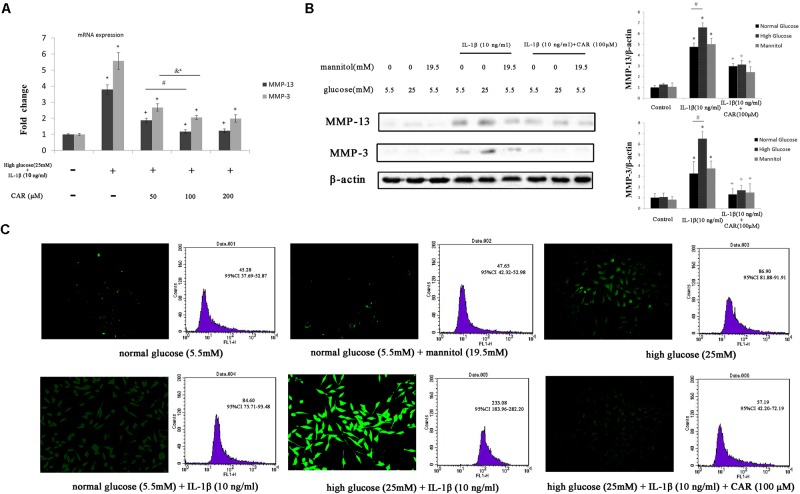
qPCR, western blotting, and ROS in FLSs. **(A)** Relative expression of MMP-3 and MMP-13 mRNA. Differences between the control group and high glucose (25 mM) with IL-1β (10 ng/ml) group were significant (^∗^*P* < 0.001); differences between the high glucose (25 mM) with IL-1β (10 ng/ml) group and different doses of CAR were significant (^+^*P* < 0.001); differences between CAR (50 μM) and CAR (100 μM) were significant (^#^*P* < 0.001 and ^&a^*P* = 0.001), but no significant differences were observed between CAR (100 μM) and CAR (200 μM). One-way ANOVA, *n* = 9; means with 95% confidence intervals. **(B)** The western blotting results of MMP-3 and MMP-13 in FLSs. Data were obtained from three separate experiments. β-actin was the internal standard. Differences between the groups were significant (^∗^*P* < 0.001, ^+^*P* < 0.001, and ^#^*P* < 0.001). One-way ANOVA, *n* = 3; means with 95% confidence intervals. **(C)** The fluorescence microscopy and flow cytometry of ROS in FLSs. *n* = 3; means with 95% confidence interval.

The relative expression levels of MMP-3 and MMP-13 mRNA are shown in **Figure 6A**. Expression of both MMP-3 and MMP-13 was higher in the high glucose (25 mM) with IL-1β (10 ng/ml) treatments than in the CG. Expression of MMP-3 and MMP-13 decreased in response to different doses of CAR compared to the high glucose (25 mM) with IL-1β (10 ng/ml) condition. No significant differences in MMP-3 or MMP-13 mRNA were observed between 100 and 200 μM CAR in the high glucose with IL-1β-induced FLSs [mRNA of MMP-3: control = 1.00 – 95% CI 0.94–1.06; high glucose (25 mM) with IL-1β (10 ng/ml) = 5.57 – 95% CI 5.05–6.09; CAR (50 μM) = 2.67 – 95% CI 2.44–2.91; CAR (100 μM) = 2.06 – 95% CI 1.94–2.17; CAR (200 μM) = 1.98 – 95% CI 1.75–2.21. mRNA of MMP-13: control = 1.00 – 95% CI 0.96–1.05; high glucose (25 mM) with IL-1β (10 ng/ml) = 3.79 – 95% CI 3.51–4.08; CAR (50 μM) = 1.87 – 95% CI 1.74–2.01; CAR (100 μM) = 1.18 – 95% CI 1.07–1.28; CAR (200 μM) = 1.22 – 95% CI 1.10–1.34]. Thus, we chose 100 μM CAR for further study.

### ROS Analysis of FLSs

We measured the production of ROS by DCFH-DA to evaluate oxidative stress in FLSs (**Figure 6C**). We replaced excess glucose with mannitol (19.5 mM) at the same concentration as a control for the hyperosmotic effects to analyze the possibility that the effect of high glucose level was due to osmotic stress. As a result, no significant difference was observed between the high mannitol group and the CG.

Reactive oxygen species production was enhanced with high glucose (25 mM) or IL-1β (10 ng/ml) stimulation separately. Interestingly, ROS production was significant enhanced with high glucose (25 mM) and IL-1β (10 ng/ml) stimulation after 24 h compared to only a single stimulation. These results emphasize the role of IL-1β and high glucose combined in the formation of ROS. Moreover, we evaluated the effect of CAR (100 μM) on ROS generation in FLSs under the high glucose (25 mM) and IL-1β (10 ng/ml) condition and noticed a significant decrease in ROS level. Furthermore, the ROS level was determined in FLSs under a fluorescence microscope after DCFH-DA staining (**Figure 6C**).

### Western Blot and Immunofluorescence Analysis of FLSs

**FIGURE 7:**
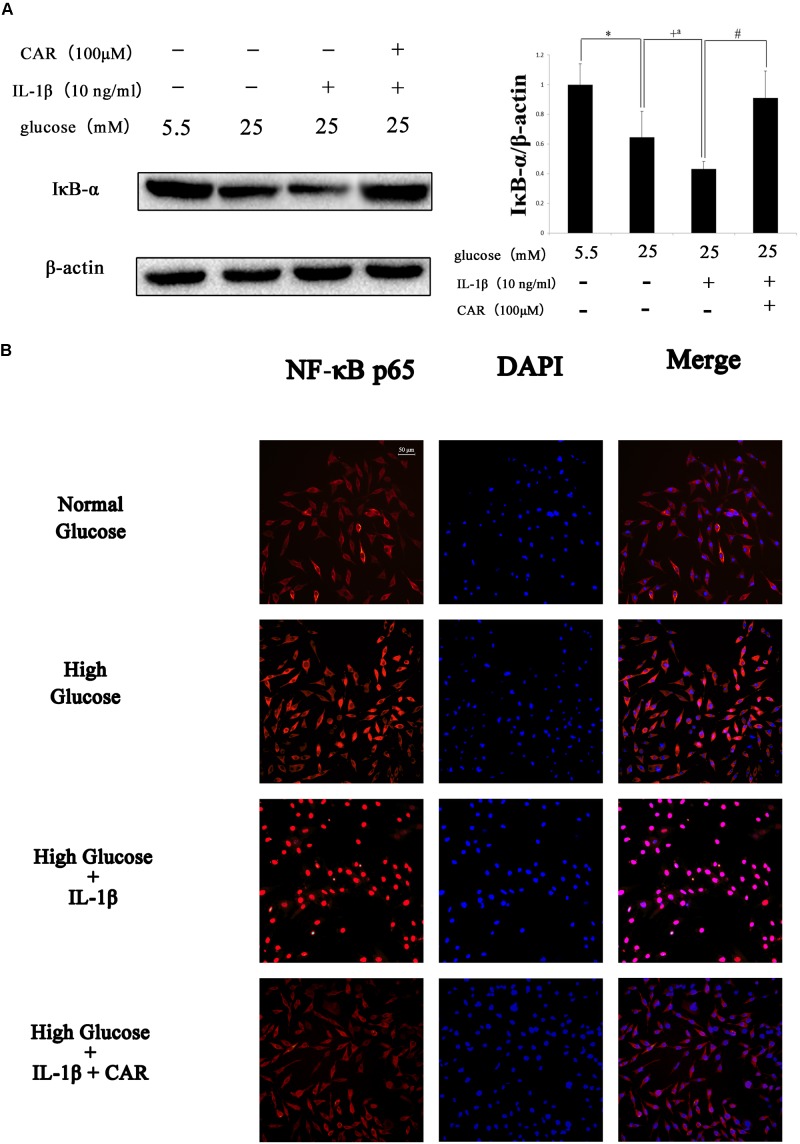
Western blotting and immunofluorescence results of NF-κB p65 in FLSs. **(A)** Western blotting results of IκB-α. Differences between the groups were significant (^∗^*P* < 0.001, ^+^*P* < 0.001, ^+a^*P* = 0.002, ^#^*P* < 0.001). One-way ANOVA, *n* = 3; means with 95% confidence intervals. **(B)** Effects of CAR on nuclear translocation of NF-κB p65 in IL-1β-induced FLSs under the high glucose condition. The FLSs were immunostained with anti-NF-κB p65 rabbit antibody (red) and visualized by confocal microcopy. Cell nuclei were defined by DAPI (blue). Scale bar, 50 μm.

We examined the effect of CAR on MMP-3, MMP-13, and IκB-α expression levels by western blot (**Figures 6B**, **7A**) and the expression of NF-κB p65 protein by immunofluorescence staining using confocal microscopy in IL-1β-induced FLSs under the high glucose condition (**Figure 7B**).

Fibroblast-like synoviocytes stimulated with or without IL-1β (10 ng/ml) and CAR (100 μM) in the presence of mannitol (19.5 mM) released quantities of MMP-3 and MMP-13 similar to those released under the normal glucose condition (5.5 mM; **Figure 6B**). Therefore, we ruled out the impact of osmotic stress under the high glucose condition.

As shown in **Figure 6B**, MMP-3 and MMP-13 protein expression increased significantly in response to IL-1β. Interestingly, MMP-3 and MMP-13 expression levels were higher in IL-1β-induced FLSs under the high glucose condition than under the normal glucose condition. Such increased inflammatory phenotype after IL-1β stimulation was corroborated by the sensitivity of the FLSs to the high glucose environment. We speculated that sustained extracellular high glucose exposure could be one of the actors in this responsiveness. However, the results show that CAR not only alleviated stimulation by IL-1β, but also ameliorated the influence of high glucose. CAR relieved the changes in IκB-α expression under high glucose (25 mM) and IL-1β (10 ng/ml) stimulation (**Figure 7A**).

As shown in **Figure 7B**, significant nuclear translocation of the NF-κB p65 protein was detected in FLSs induced by high glucose (25 mM) and IL-1β (10 ng/ml) when compared with the CG. Furthermore, **Figure 7** also indicates that CAR suppressed nuclear translocation of NF-κB p65, which was used to confirm our hypothesis that CAR exhibited therapeutic effects by suppressing nuclear translocation of NF-κB p65.

## Discussion

The principal findings of the present study were: (i) T2DM caused knee OA and oral supplementation with CAR at doses of 0.3 and 0.9 g/kg partially alleviated T2DM-induced OA by reducing cartilage surface erosion, matrix loss, and inflammation of the synovium; (ii) the FLSs showed increased responsiveness to IL-1β-induced inflammation under the high glucose condition; and (iii) CAR suppressed the inflammatory response in IL-1β-induced FLSs under the high glucose condition via the ROS/NF-κB pathway.

To study the association between T2DM and progression of OA, we investigated the implications of T2DM in the development of OA using a high-fat diet and low dose STZ-treated model. These rats exhibit metabolic disturbances similar to those observed in humans with T2DM; thus, they are a representative model to study diabetes and associated metabolic complications (Shi et al., 2011). In the first 4 weeks of high-fat diet, the weight of rats did increase significantly. However, after injection of STZ, there was no significant change in the body weight of T2DM rats, which were even lower than CG group (**Figure 3B**). Therefore, the effect of weight gain is not obvious for OA compared to T2DM, which supports the findings of previous reports (Courties et al., 2015; Cicuttini and Wluka, 2016). T2DM is related to systemic low-grade chronic inflammation characterized by abnormal cytokine production and activation of a network of inflammatory signaling pathways (Hotamisligil, 2008; Courties et al., 2015). Our results show that rats with T2DM developed more severe OA-like changes, which caused histological changes, including cartilage surface erosion, matrix loss, and inflammation of the synovium. Notably, inflammation of the synovium and IALF are found in the joint. Joint inflammation is accompanied by increases in MMP-13 and MMP-3, predominantly in the synovium. Taken together, these results support that abnormal glucose metabolism and the inflammatory response accelerate experimental OA in rats with T2DM. Thus, abnormal glucose metabolism and the inflammatory response could be the mechanisms responsible for T2DM-induced OA.

The therapeutic potential of CAR is reflected by its antioxidant and anti-inflammatory capacities (Shi et al., 2011); thus, we evaluated whether an oral CAR treatment could confer protection to rats with T2DM-induced OA. Our results show that CAR decreased systemic inflammation in rats with T2DM-induced OA which was monitored by serum IL-1β and TNF-α. In addition, the oral CAR treatment dramatically ameliorated synovitis, as demonstrated by the expression of IL-1β in the synovium and the concentration of inflammatory cytokines in IALF. Pro-inflammatory mediators produced by FLSs, such as cytokines, degrade cartilage, and CAR, alleviated cartilage surface erosion according to the histological evaluation.

Low-grade inflammation and hyperglycemia are observed in T2DM. Growing evidence suggests that targeting FLS-mediated synovial inflammation and invasion may be a new therapeutic avenue for OA. MMP-3 and MMP-13, which degrade the extracellular matrix of cartilage, play a vital role in the occurrence, development, and progression of OA (Nair et al., 2012; Ping et al., 2017). FLSs express MMPs, as well as a variety of surface adhesion molecules. Thus, we investigated the expression of MMPs by FLSs in response to high glucose and IL-1β. We found that IL-1β increased the synthesis of MMP-3 and MMP-13 in FLSs *in vitro*. Intriguingly, we also discovered that MMP-3 and MMP-13 expression levels increased markedly when IL-1β-induced FLSs were cultured under the high glucose condition compared to those cultured under the normal glucose condition. However, no significant differences were observed between the normal and high glucose environment without IL-1β stimulation. Thus, it was a particularly noteworthy finding that the enhanced pro-inflammatory response under the high glucose environment was related to sensitization of FLSs according to their responsiveness to IL-1β-induced inflammatory stress.

The CAR treatment had a therapeutic effect on MMP-3 and MMP-13 production in IL-1β-induced FLSs under the normal and high glucose conditions, suggesting that CAR specifically reversed the inflammatory response. We speculate that the therapeutic effect of CAR is related to ameliorating the inflammatory response and sensitizing FLSs under the high glucose condition. Moreover, CAR abolished the additive pro-inflammatory effect of high glucose in IL-1β-induced FLSs.

**FIGURE 8:**
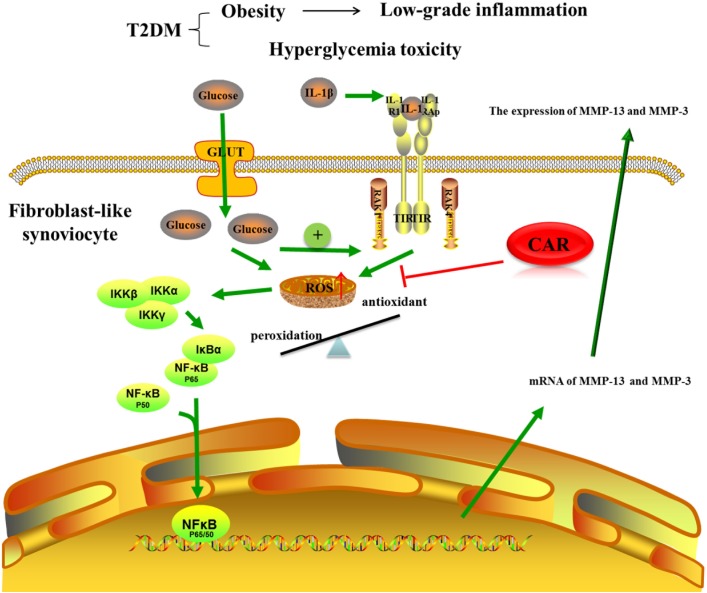
The mechanism of CAR on IL-1β-induced FLSs in a high glucose environment. CAR alleviated MMP-3 and MMP-13 expression by decreasing ROS content and suppressing nuclear translocation of NF-κB p65 in IL-1β-induced FLSs under a high glucose environment.

Increased activity of the ROS pathway, which occurred when FLSs were induced by IL-1β in the high glucose condition, is an important pathogenic factor in diabetic complications. Excessive production of ROS damages protein, lipids, nucleic acids, and matrix components (Chuang et al., 2014). Mitochondria are vital for cellular bioenergetics and regarded as the major cellular site for ROS production. Overwhelming data suggest that reactive lipid mediators generated from this process are biomarkers for oxidative stress and important players for mediating a number of signaling pathways (Zhong and Yin, 2015). Actually, FLSs from an OA joint can produce large amounts of ROS in response to biochemical stimuli. The FLSs behaved differently after 24 h of the high and normal glucose conditions induced by IL-1β. In the pro-inflammatory and high glucose condition, the mitochondrial respiratory chain would be more activated, which would increase mitochondrial production of ROS. To investigate the link between the targets potentially responsible for these effects, we evaluated activity of the NF-κB pathway, a key signaling pathway that regulates the cellular inflammatory response. Nuclear factor (erythroid-derived 2)-like 2 (Nrf2) also plays a prominent role in orchestrating glucose metabolism (Mitsuishi et al., 2012). It is now clear that robust NF-κB and Nrf2 activity is essential for maintaining coordinated cellular responses to resolve the inflammatory status of the cell/tissue (Wardyn et al., 2015). But the relationship between Nrf2 and NF-κB pathway on IL-1β-induced FLSs in a high glucose environment needs further investigation. Our study shows that ROS produced by mitochondria promoted nuclear translocation of NF-κB p65, which activated the NF-κB signaling pathway (**Figure 8**). Thus, suppressing excess production of ROS could be of benefit in the prevention and management of T2DM-induced OA. Ultimately, we treated FLSs with CAR, which had promising results decreasing ROS and suppressing nuclear translocation of NF-κB p65.

It has been reported that T2DM dramatically affects health outcomes, including articular cartilage and synovial function. Thus, the mechanism of T2DM and OA needs further investigation. Based on these concepts, we demonstrated that T2DM activates systemic inflammatory mediators and the high glucose condition increases sensitization of FLSs to IL-1β-induced inflammatory stress via the ROS/NF-κB signaling pathway. Our findings suggest that CAR could provide a safe alternative to current pharmacological therapies for T2DM-induced OA.

## Conclusion

In conclusion, our findings demonstrate that T2DM can cause knee OA, and an oral CAR treatment partially inhibited the development of OA by reducing cartilage surface erosion, matrix loss, and inflammation of the synovium. Moreover, FLSs from rats under a high glucose condition were more reactive to pro-inflammatory stress involving oxidative stress and the NF-κB pathway than those under a normal glucose condition. CAR ameliorated the changes in FLSs induced by IL-1β under the high glucose condition. These results emphasize the potential therapeutic value of oral CAR to treat T2DM-induced OA.

## Author Contributions

YY and LB contributed to the conception and design. YY, YW, XZ, and YG involved in the animal experiment. YY, YW, and HZ involved in cell cultures. All authors contributed to acquisition and analysis of data, revising the manuscript critically for important intellectual content, and approved the manuscript for publication. YY, YW, and YK involved in statistical analysis and manuscript preparation. LB conceived the final approval of the version to be submitted and contributed to obtaining the funding.

## Conflict of Interest Statement

The authors declare that the research was conducted in the absence of any commercial or financial relationships that could be construed as a potential conflict of interest.

## Supplementary Material

FIGURE S1 FLSs were isolated from the knee joints of rats. Representative immunofluorescence image of FLSs stained with vimentin (green). Nuclei were stained with DAPI (blue). Scale bar, 50 μm.
