# Supplementary figures and images for: Carnosine Prevents Type 2 Diabetes-Induced Osteoarthritis Through the ROS/NF-κB Pathway
Source: Front Pharmacol. 2018 Jun 6;9:598. doi: 10.3389/fphar.2018.00598 (PMC5997783; doi:10.3389/fphar.2018.00598)

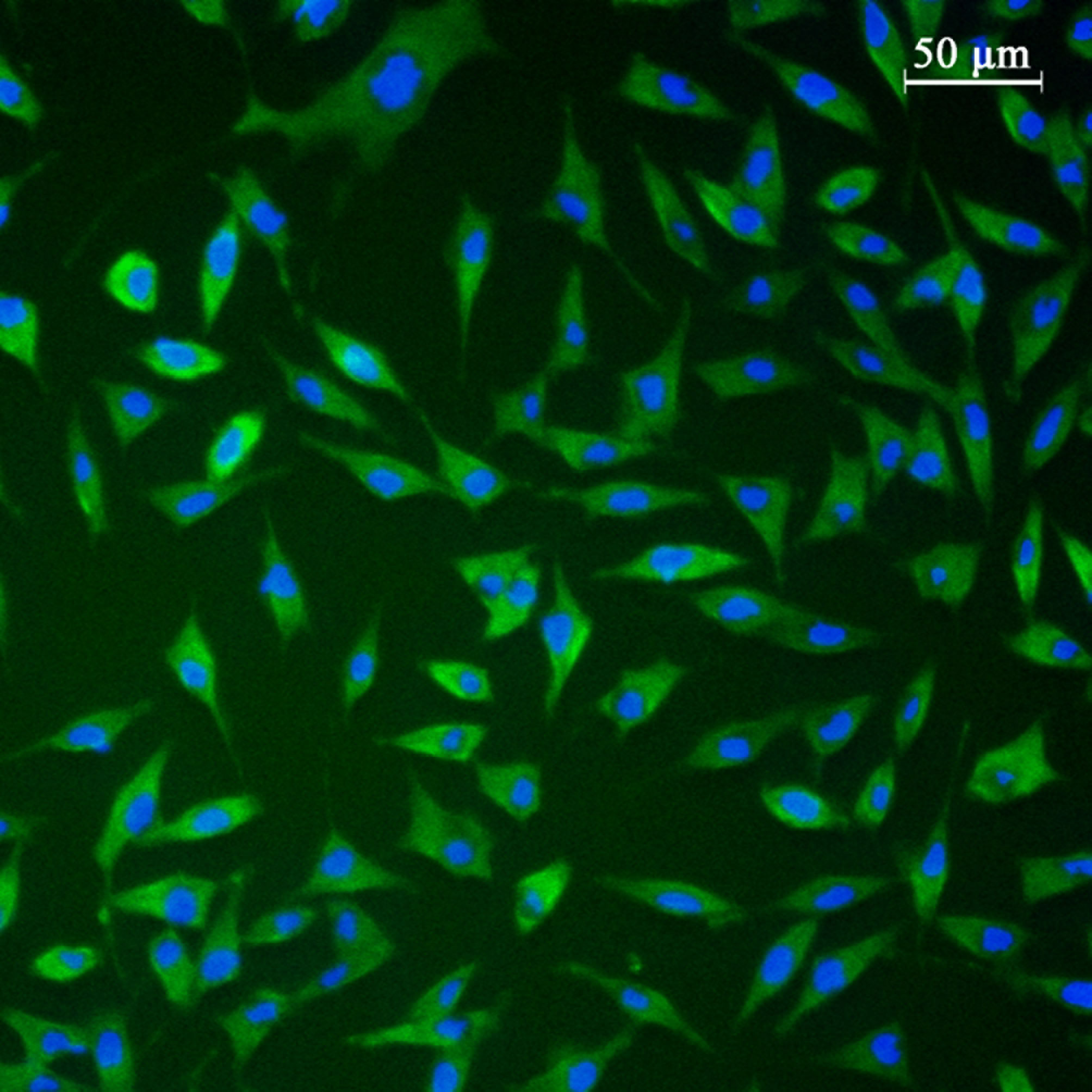

Supplement: FIGURE S1 — FLSs were isolated from the knee joints of rats. Representative immunofluorescence image of FLSs stained with vimentin (green). Nuclei were stained with DAPI (blue). Scale bar, 50 μm. [file Image_1.TIF]
